# First Detection of Human ST131-CTX-M-15-O25-B2 Clone and High-Risk Clonal Lineages of ESBL/pAmpC-Producing *E. coli* Isolates from Diarrheic Poultry in Tunisia

**DOI:** 10.3390/antibiotics10060670

**Published:** 2021-06-04

**Authors:** Ahlem Jouini, Amira Klibi, Imen Elarbi, Meriem Ben Chaabene, Safa Hamrouni, Oussema Souiai, Mariem Hanachi, Abdeljelil Ghram, Abderrazak Maaroufi

**Affiliations:** 1 Laboratory of Epidemiology and Veterinary Microbiology, Group of Bacteriology and Biotechnology Development, Institut Pasteur de Tunis, University of Tunis El Manar, Tunis 2092, Tunisia; klibi.amira@yahoo.fr (A.K.); Imen.Larbi@pasteur.tn (I.E.); chaabene.meriem@gmail.com (M.B.C.); safa.hamrouni@pasteur.tn (S.H.); Abdeljelil.Ghram@pasteur.tn (A.G.); Abderrazak.Maaroufi@pasteur.tn (A.M.); 2 Laboratory of Bioinformatics, Biomathematics and Biostatistics-LR16IPT09, Institute Pasteur de Tunis, University of Tunis El Manar (UTM), Tunis 2092, Tunisia; oussema.souiai@pasteur.tn (O.S.); mariem.hanachi@gmail.com (M.H.); 3 Faculty of Sciences of Bizerte, University of Carthage, Jarzouna-Bizerte 7021, Tunisia

**Keywords:** ESBL, ST131, clonal lineages, pAmpC, diarrheic poultry, pathogenic bacteria

## Abstract

Circulation of a multi-resistance clone of bacteria associated with genetic elements in diseased animals constitutes a global public health problem. Our study focused on the characterization of the support of ESBL in cefotaxime resistant *E. coli* (CTX^R^) isolates recovered from poultry with diarrhea, analysis of their clonal lineage, and virulence-associated genes. The study was carried out on 130 samples of chickens with diarrhea, collected in 2015 from poultry farms in Tunisia. Isolates of 20 CTX^R^ *E. coli* strains were identified as ESBL and AmpC β- lactamase producers. The following β-lactamase genes (number of isolates) were detected: *bla*_CTX-M-15_+ *bla*_OXA1_ (4), *bla*_CTX-M-15_ + *bla*_OXA1_ + *bla*_TEM-1b_ (2), *bla*_CTX-M-1_ + *bla*_TEM-1b_ (9), *bla*_CTX-M-1_ (2), *bla*_CMY2_ + *bla*_TEM-1b_ (3). Six *E. coli* harboring *bla*_CTXM-15_ were allocated to ST131-B2-O25b-; six and three *bla*_CTX-M-1_ were grouped in ST155, ST10, and ST58, respectively, related to the phylogroup D and A. The *qnrB* gene, the variant *aac(6′)-Ib-cr,* and the class 1 integrons with different gene cassettes, were detected amongst our 20 isolated strains, which were classified as ExPEC and aEPEC. Our findings highlighted the emergence of the human pandemic ST131-CTX-M-15-O25-B2 clone and the high risk of such clonal lineage strains in diarrheic poultry, in Tunisia, which could constitute a risk of their transfer to healthy animals and humans.

## 1. Introduction

*Escherichia coli* is a common microorganism of the intestinal microbiota of humans and animals and may be involved as an opportunistic and zoonotic pathogen for humans and animals [1]. Pathogenic *E. coli* in food-producing animals can cause serious economic losses in livestock related to bovine mastitis, avian colibacillosis, and diarrhea. Diarrhea is considered a major health concern in poultry and contributes significantly to economic losses in the poultry industry in Tunisia and worldwide since it leads to mortality, seizures, and reduction of performances in slaughterhouses [2]. Bacterial diarrhea or dysbacteriosis, a non-specific bacterial enteritis, is another highly cited cause of wet excreta. Dysbacteriosis is due to the imbalance of the intestinal microbiota caused by several non-infectious and infectious factors such as coccidia and *Clostridium perfringens*. Dysbacteriosis may also be a result of an abrupt change in feed or other stress factors, which upset the usual microbial balance in the intestine. Pathogenic bacteria, like *Escherichia coli*, *Campylobacter jejuni*, and *Spirochaetes*, as well as several viruses, are known to be involved as causative agents of avian diarrhea. Indeed, *E. coli* often infects poultry via the gastrointestinal tract and causes diarrhea and septicemia. Pathogenic strains have been divided into extraintestinal pathogenic *E. coli* (ExPEC) and intestinal pathogenic *E. coli* (diarrheagenic; DEC). Enteropathogenic *E. coli* (EPEC) is one of the six pathotypes of diarrheagenic *E. coli* that produce severe infant diarrhea [3]. EPEC can produce attaching and effacing lesions, which are characterized by intimate bacterial attachment coding *eae* gene and classified as typical EPEC (tEPEC) and atypical (aEPEC). This classification depends on the presence or absence of the bundle forming pilus A (*bfpA*) gene. The reservoir for (tEPEC) is considered in humans; in contrast, (aEPEC) strains are more prevalent in animals [4].

Over the last few years, there was a worrisome global health problem concerning extended spectrum-beta-lactamase (ESBL)-producing *E. coli* in human and animal infections, in both hospital and livestock settings [1]. ESBL-producing *E. coli* isolates, especially those producing ESBL of CTX-M type, have increasingly been reported worldwide over the last two decades [1,2]. Based on the diversity of ESBL enzymes among *E. coli* isolates from African livestock, those belonging to the CTX-M-1 group have been reported to be more frequent than the classical SHV- and TEM- ESBL enzymes [2,5]. CTX-M-1 and CTX-M-14 enzymes are so far the most common ESBLs in *E. coli* isolated from livestock, especially poultry and food products of animal origin [5,6]. Nevertheless, the CTX-M-15 enzyme is the most common enzyme largely disseminated in the clinical settings, as well as in the community with few exceptions, worldwide [7]. The successful spread of the CTX-M-15 enzyme is largely attributed to its association with the highly virulent pandemic lineage ST131-O25b-B2 *E. coli*. This clonal lineage is a major cause of severe human extraintestinal infections, including community-acquired urinary tract infections [8]. In addition, it has also been reported in companions and non-companion animals with human contact, in some countries [9]. Indeed, a large multinational European report showed that 6% of ESBL- producing *E. coli,* isolated from various companion animals, belonging to *E. coli* ST131 [9]. This clonal lineage has also been reported at low frequencies in livestock, food products of animal origin, vegetables, as well the environment [9].

The scarce occurrence of *E. coli* ST131 favors the hypothesis that humans, rather than the companion and food-producing animals, serve as the primary reservoir of such clone [9].

There are a few reports of clinical infections of animals caused by *E. coli* ST131, especially in poultry livestock. Therefore, surveillance of foodborne pathogens, drug resistance, and virulence determinants are important for conducting epidemiological analysis of such factors, designing risk management, and strategic control of poultry farming; these data being highly relevant to public health.

This study aimed to characterize the support of ESBL in cefotaxime resistant *E. coli* (CTX^R^) isolates recovered from poultry with diarrhea and analyze their clonal lineages, as well as the presence of virulence-associated genes to find out the potential threat to food safety and risk to public health of circulating pandemic multi-resistance clone in chickens.

## 2. Results

### 2.1. E. coli Recovery from Poultry Samples

Cefotaxime-resistant isolates (CTXR) were recovered in 20 out of 130 (15.5%) analyzed samples, with 11/80 (13.75%) from feces and 9/50 (18%) from tracheal swabs. Details of pathogen isolation are presented in Table 1 and Figure S1 (Supplementary material). Only positive fecal and tracheal samples are presented. Biochemical and molecular identification showed that the 20 isolates were assigned to *E. coli* species harboring the specific gene *iudA*. Fourteen recovered *E. coli i*solates from fecal and tracheal animal samples, collected from a different department, were labeled as (strain/chicken n°): EC1, EC2/C1; EC3, EC4/C5; EC5, EC7/C9; EC9, EC15/EC6; EC10, EC11/C4; EC13, EC14/C8; EC18, EC19/EC10.

### 2.2. Antibiotic Resistance Rates

The occurrence of antibiotic resistance in the 20 cefotaxime-resistant isolates is presented in Table 1 and Figure S1. No isolate had resistance to imipenem or ertapenem. High rates of resistance were observed for sulfonamides (80%), tetracycline (75%), nalidixic acid (60%), and trimethoprim/sulfamethoxazole (55%). However, medium and low frequency of resistance were observed for streptomycin (30%), ciprofloxacin (25%), chloramphenicol (10%), tobramycin (5%) and gentamicin (5%). All isolates except one (*E. coli* 19) showed multi-drug resistance phenotypes, including resistance to at least three families of antimicrobial agents. In addition, the 14 *E. coli* strains isolated from fecal and tracheal samples of the same animal have different antibiotic resistance patterns.

According to the Double Disc Synergy Test (DDST), 17 and three *E. coli* isolates were ESBL- and AmpC-producers, respectively (Table 1). Consequently, the prevalence of ESBL-positive *E. coli* isolates in collected samples was 13%, and the prevalence of AmpC phenotype was 2.3%.

### 2.3. Characterization of β-Lactamases Gene and Genetic Environment of bla_CTXM_ Genes

The β-lactamases genes detected in the 20 cefotaxime-resistant *E. coli* isolates are summarized in Table 1. Among the 17 ESBL-positive isolates, 11 and 6 contained *bla*_CTX-M-1_ either *bla*_CTX-M-15_ genes, respectively; *bla*_OXA-1_ and *bla*_TEM-1b_ genes being detected in 6 and 11 isolates, respectively, mainly in association with *bla*_CTXM-1_ and *bla*_CTXM-15_ genes. The following gene associations were detected (number of isolates): *bla*_CTX-M-15_ + *bla*_OXA-1_ (*n* = 4), *bla*_CTXM-15_ + *bla*_OXA-1_ + *bla*_TEM-1b_ (*n* = 2), *bla*_CTXM-1_ + *bla*_TEM-1b_ (*n* = 9), *bla*_CTX-M-1_ (2). The three AmpC-producing isolates harbored the *bla*_CMY-2_, which was associated with the *bla*_TEM-1b_ gene in all of them.

The region surrounding the *bla*_CTX-M_ and the *bla*_CMY-2_ detected genes are shown in Table 1. The *orf*477 sequence was present downstream of *bla*_CTX-M-15_ and *bla*_CTX-M-1_ genes, in all positive isolates. In the three *bla*_CMY-2_-positive isolates, the downstream region could not be identified despite many attempts of PCR amplification. On the other hand, the IS*Ecp1* sequence was identified upstream of the *bla*_CTX-M_ gene, in eleven isolates and all *bla*_CMY-2_-positive isolates. Interestingly, the *IS*26 flanked a partially truncated IS*Ecp1* element in three *bla*_CTX-M-15_ positive *E. coli* isolates.

### 2.4. Characterization of Integrons and Resistance Mechanism to Non β-Lactam Antibiotics

The presence of class 1 integrons was demonstrated in eleven isolates (55%) and three gene cassette arrangements were identified in their variable regions (number of strains): *dfrA17* + *addA5* (6), *dfrA1* + *aadA1* (3), *dfrA12* + *aadA2* (2). Class 2 integrons were not detected in ours isolated strains. The 3′conserved sequence (*qac*EΔ1-*sul*1) was detected in all *intI*1-positive isolates.

A variety of resistance genes to non β-lactam antibiotics and located outside the integrons were observed in 19 out of 20 isolates (Table 1, Figure S1). The *qnr B* and *aac(6′)–Ib-cr* genes were detected in four and six quinolone-resistant isolates, respectively. Tetracycline resistance was encoded by *tet (A)* and *tet (B)* genes in eleven and five isolates, respectively. The *sul2* and *sul3* genes were detected in 13 and 4 isolates, respectively, and two strains harbored both genes.

### 2.5. Phylotyping and Virulence Genotyping of E. coli

Phylogeny analysis revealed that our isolates belong to phylogroups D (9 isolates), B2 (6 isolates), B1 (3 isolates), and A (2 isolates). Overall, the 20 ESBL/AmpC-producing *E. coli* isolates (100%) carried at least the two virulence genes (*fimA* and *cnf1*) under investigation (Table 2). However, *eae, aer*, *papC,* and *papG* genes were detected in six, four, ten, and six isolates, respectively. The six ST131-B2- *E. coli* contained five of the eight virulence genes studied and were all affiliated to the ExPEC pathovar. In addition, six ESBL-producing *E. coli* isolates belonging to phylogroup D were assigned to the aEPEC pathovar. The remaining eight isolates were attributed to the ExPEC pathovar, according to their virulence-gene contents (Table 2, Figure S1).

### 2.6. Serotyping and Molecular Typing of ESBL—E. coli Strains

The analysis of MLST showed six sequence types (STs), being 6 ST131, 5 ST155, 3 ST10, 3 ST58, 2 ST2179 and 1 ST1011. Six CTX-M-15 producing *E. coli* type ST131-B2 belonged to the serotype O25b but none of them presented the serotype O25a or contained the new described operon *afa*/*dra*. Taken together, the combination of both typing methods showed the following classification, according to the type of produced enzyme: (i) CTX-M-15-positive isolates: ST131-B2 (*n* = 6), (ii) CTX-M-1-positive isolates: ST155-D (*n* = 5) + ST10-D/A (*n* = 2/1) + ST58-D/A (*n* = 2/1), (iii) CMY-2-positive isolates: ST2179-B1 (*n* = 2) + ST1011-B1(*n* = 1).

### 2.7. Phylogeny of Resistance, Virulence Genes and Clone in E. coli Strains

Figure S1 illustrates the relationships between sample origin, resistance and virulence genes, sequence type, and pathovars of ESBL/AmpC producing *E. coli* isolates.

Noteworthy, all ESBL producing *E. coli* strains harboring the sequence type ST131-CTX-M-15 clustered together and showed the same virulence determinants, pathovar, and integron 1 carried the same arrangement of gene cassettes; while only one ST131-*E. coli* strain did not contain integrons. In addition, all isolated ST131-*E. coli* did not harbor the same resistance genes detected outside integrons (Table 1, Figure S1). Conversely, the CTX-M-1 *E. coli* producing strains with different sequence types (ST) (ST155, ST58, ST10), different resistance, virulence genotyping, and pathovars were clustered together, except the strain EC15 that is clustered alone and affiliated to the ST155. In addition, the strains EC5 and EC9 recovered from tracheal samples lost their *qnrB* gene in a fecal sample collected from the same animal (EC7, EC15).

## 3. Discussion

To the best of our knowledge, there is no published information regarding clonal lineages and virulence factors of ESBL/pAmpc producing *E. coli* from diarrheic chickens, in Tunisia. Only very limited data on the prevalence and molecular characterization of ESBL *E. coli* producing strains isolated from healthy animals, food-producing animals, and chickens suffering from colibacillosis, are available from Africa and Tunisia [2,10].

For this, 130 avian samples collected from diarrheic chickens of a commercial farm, were analyzed and 20 (15.4%) cefotaxime-resistant *E. coli* isolates from fecal and tracheal swabs were detected. Among them, 17 were ESBL and three pAmpC producers. These findings were similar to few published studies reporting the presence of ESBL-*E. coli* producing isolates in clinical chickens suffering from colibacillosis, in Tunisia, and showing much higher frequency (58.3%) of ESBL-producers than found in our study [11]. In addition, the percentage of ESBL-*E. coli* isolates reported in our study (15.5%) were higher than that found, respectively, in feces and internal organs in chickens with colibacillosis (4.1%, 0%) [12,13]. It is important to note that 14 ESBL/AmpC-producing *E. coli* strains detected in this study were isolated from fecal and tracheal samples from the same chickens with diarrhea in different departments of the farm. It seems that these strains are infectious agents present in the visited farm. The spread of multi-resistance ESBL/AmpC producing *E. coli* strains, in livestock in Tunisia, could be related to the selection pressure that abusive antimicrobial use in animals might generate. The widespread use of different classes of antibiotics as growth factors or for therapeutic and preventive control in poultry is not very documented and remains largely unregulated in African countries [14].

In the present study, it was shown that *bla*_CTXM-1_ associated or not to *bla*_TEM1-B_ is the most ESBL gene detected in diarrheic chickens. Indeed, in Tunisia, the *bla*_CTXM-1_ gene was frequently detected in *E. coli* isolates from healthy poultry and pet [2,15] and accounted as one of the major ESBL genes reported in animals worldwide, especially in African livestock [2]. Surprisingly, aside from the ESBL enzyme CTX-M-1 characterized in *E. coli* isolates, the *bla*_CTX-M-15_ gene was detected in six isolates. CTX-M-15 has been frequently reported from human ESBL-producing *Enterobacteriaceae* in Africa, including Tunisia, and in other regions of the world [2,16,17]. In addition, it appears more prevalent in cattle than in other animal livestock [12]. CTX-M-15 was reported in bovine mastitis and feces of healthy poultry and sheep, in Tunisia [18,19] and from internal organs of chickens with septicemia and colibacillosis, in Algeria [2].

In the same way, the present study constitutes the first description of *bla*_CMY-2_-positive *E. coli* strains, isolated from poultry with diarrhea in Tunisia. However, this β-lactamase variant was also identified among commensally *E. coli* from healthy chickens, in Tunisia [5,20], Algeria [21], septicemic broilers in Egypt [10], healthy humans, pets and food animals, worldwide [18,22].

Dissemination of ESBL/AmpC-encoding genes has been largely linked to the spread of some particular competitive international high-risk clones; this emergence was also observed for our studied isolates. Indeed, the international high-risk clones ST131, ST155, ST10 were found in six and three ESBL-producing isolates, respectively. The ST131 isolates were CTX-M-15 producers and were associated with the serotype O25b, and assigned to the phylogenetic group B2. To the best of our knowledge, this study is the first to reveal the presence of the pandemic high-risk human lineage CTX-M-15-B2-O25b-ST131 *E. coli* from diseased chickens in Tunisia. Furthermore, such isolates were the major global clone in human CTX-M-15-producing *E. coli* strains. They have also been identified at low frequencies in healthy swine in Tanzania [23], the blood of septicemic broilers in Egypt [10], healthy poultry and pig in Spain, as well as in companion and non -companion animals (seagulls and rates both of which have close contact with human) [19,24]. In Tunisia, many studies have reported the occurrence of ST131 in human settings (hospitalized patients with urinary infection, newborns, patients in intensive care units, clinical samples [6,25,26,27]. The wide spread of the ST131 clone is mainly linked to competitiveness and armament by genes encoding antibiotic resistance and virulence factors. In addition, all these isolates harbored the same profile of virulence genes (*cnf1-fimA-papG-aer-papC*) and were assigned as ExPEC. They were quinolone-resistant and trimethoprim/sulfamethoxazole resistant coded, respectively, by variant *aac(6′)-Ib-cr* and integrons class 1. These results are similar to others reporting worldwide that fluoroquinolone and trimethoprim/sulfamethoxazole resistance are significant markers of ST131 clone and the “dual substrate” aminoglycoside-modifying enzyme *aac(6′)-ib-cr* contributes to quinolone resistance via acetylation [24].

The presence of the clone ST155 (five isolates) and ST10 (3 isolates) harboring the *bla*_CTXM-1_ gene was similar to other studies reporting that these lineages are associated with multi-drug resistance, mainly CTX-M variants [28]. It is worthy to note that ST155 has commonly been reported in healthy poultry in Tunisia and Africa [2,6,28]. It appears to be associated with a zoonotic risk, suggested by some studies [28]. In addition, the ST10 was highly distributed among various animal livestock and humans in Africa [2]. In Tunisia, it was also reported in *E. coli* from food origins [29] and recently considered as the most disseminated among commensal *E. coli,* worldwide [30]. The ST10 and ST155 clones were detected in Algerian farmlands and associated with colistin resistance [31]. These findings of sequence type ST2179 and ST1011 related to CMY-2, in our collection, were also reported from healthy chickens in Tunisia and Algeria [2,20].

The IS*Ecp1* insertion sequence has been identified upstream *bla*_CTX-M_ and *bla*_CMY-2_ genes and, interestingly, in three isolates, *IS*26 flanked a partially truncated IS*Ecp1* element. These results suggested a crucial role of IS*Ecp1*-like sequences in the mobilization and expression of *bla*_CTX-M_ genes [1,7].

The detection of class 1 integron in eleven CTX-M positive *E. coli,* with a different arrangement of gene cassettes *dfr* and *aad,* conferred trimethoprim and streptomycin resistance, respectively. This finding agreed with many studies from different origins in Tunisia and Africa [2,32].

Furthermore, the observed quinolone resistance encoded by *qnrB* plasmid could be due to the overuse of this antibiotic family in chickens to treat or prevent bacterial infections in poultry farms. This result was similar to previous studies interested in livestock infections [33]. The presence of *qnrB* plasmid in *E. coli* strains isolated from tracheal samples and not recovered from fecal samples of the same animal, showed that these strains could lose this plasmid during their passage through the digestive tract.

The presence of ESBL/AmpC *E. coli* producing strains harbored CTX-M-1 in the same cluster and assigned to different clonal lineages (ST155, ST58, ST10) could be due to the horizontal transmission of plasmids bearing these ESBL genes. In this sense, a few molecular studies in Tunisia described that CTXM-1 was broadly disseminated in healthy poultry associated with Inc1/ST3 plasmids [12,34]. Nevertheless, the phylogenetic presence of ESBL producing *E. coli* strain EC15/ST155 harbored CTX-M-1 in distant clusters suggested that the ESBL gene was carried by another type of plasmid. The detection of ESBL *E. coli* ST131-CTX-M-15-B2 classified in the same cluster could be due to its association with the same replicon plasmid. This finding agreed with studies in Africa [2].

Phylogenetic analyses have shown that the majority of ours strains are assigned to the phylogroups B2 and D with three to five virulence factors; only three strains being affiliated to the phylogroup B1. These findings were in concordance with those of other studies that report pathogenic strains producing enteropathogenic (EPEC) and extraintestinal (ExPEC) infections mainly belonging to the phylogroups B2 and D [35].

According to the content of genes encoding virulence factors, six isolates were classified as atypical EPEC owing to the presence of intimin and the absence of bundle forming pili encoding *eae* and *bfp* genes, respectively. The results corroborated with other studies describing animals, especially poultry, as the major reservoir of EPEC, which constitutes the major zoonotic enteropathogenic bacteria [4,36]. Furthermore, the remaining *E. coli* isolates were assigned to the ExPEC group with the presence of virulence genes specific to this pathovar [32]. ExPEC is widespread amongst animal reservoirs and causes several infections [3,37]. Besides, our strain collection harbored the cytotoxic necrotizing factor (*cnf1*) and type 1 fimbria (*fimA)*; these virulence factors are reported as the most important virulence factor genes found in *E. coli* strains with P fimbria (*pap)*, fimbrial adhesion I (*afa*) and aerobactin (*aer)* [38]>.

## 4. Materials and Methods

### 4.1. Sampling

Eighty fecal and 50 tracheal samples were collected from commercial chickens with diarrhea, reared in a farm in the region of Sidi Thabet (North of Tunisia), in 2015. The farm contained 13 broiler houses, each accommodating 10,000 birds and from which cloacal and tracheal swabs were taken by the veterinarian. All samples, collected from diseased animals that show clinical symptoms such as dyspnea, reduced appetite, and diarrhea, were directly transferred under cold conditions to the laboratory and immediately processed. Table 1 presents the details of sampling.

### 4.2. Isolation and Identification

The swabs were suspended in 5 mL of brain-heart broth (Biolife, Italy) and incubated for 24 h at 37 °C. After serial dilutions, 100 µL of the bacterial suspension were streaked onto MacConkey (Biolife, Italy) agar plates supplemented with cefotaxime (2 μg/mL) and imipenem (1 µg/mL). Colonies with typical *E. coli* morphology were selected (one colony per sample) and identified by classical biochemical methods for catalase, oxidase, indole, citrate, and urease characters. The *E. coli* isolates were submitted to molecular identification by species-specific PCR of the *uidA* gene [39]; *E. coli* strain ATCC 25922 was used as a control.

### 4.3. Antimicrobial Susceptibility Testing

Antimicrobial susceptibility testing was conducted on Mueller-Hinton agar (Biolife, Milano, Italy) plates using the agar disk diffusion method, according to Clinical and Laboratory Standards Institute (CLSI) criteria [40]. The tested antibiotics were (µg/disc): ampicillin (10), ticarcillin (75), amoxicillin/clavulanic acid (20 + 10), cefoxitin (30), ceftazidime (30), cefotaxime (30), Imipenem (10), ertapenem (10), gentamicin (10), tobramycin (10), streptomycin (10), nalidixic acid (30), ciprofloxacin (5), sulphonamides (200), trimethoprim/sulfamethoxazole (1.25 + 23.75), tetracycline (30), and chloramphenicol (30). *E. coli* strain ATCC 25922 was used as a control strain. A screening test for the detection of ESBLs was carried out by the (DDST), according to the CLSI criteria [40] and using previously reported strain collection [39].

### 4.4. Characterization of Beta-Lactamase Genes and Genetic Environment of bla_CTX-M_ Genes

The genes encoding TEM, SHV, OXA-1, CTX-M, CMY type beta-lactamases in all ESBL-positive isolates were analyzed by specific PCRs, as previously reported [39]. All obtained amplicons were sequenced on both strands and sequences were compared with those included in the GenBank database and the Lahey clinical website (http://www.Lahey.org/Studies/webt.html, accessed on 17 June 2019), to identify the specific type of beta-lactamase gene [41]. The IS*Ecp1*, IS*26,* and *orf477* sequences surrounding the *bla*_CTX-M_ and *bla*_CMY_ genes were analyzed by PCR, using previously described primers and conditions [16]. All obtained amplicons were sequenced for confirmation. Positive and negative control strains, from the collection of the Laboratory of Epidemiology and Veterinary Microbiology, at Institute Pasteur of Tunis, were included in all the PCR assays [16,39].

### 4.5. Characterization of Integrons and Resistance Mechanisms to Non-β-Lactam Antimicrobial Agents

The presence of genes associated with the resistance to tetracycline (*tetA* and *tetB*), sulphonamides (*sul1*, s*ul2*, and *sul3*), streptomycin (*strA* and *strB*), and quinolone (*qnrA*, *qnrB*, *qnrS* and *aac(6′)-Ib-cr*), was determined for all resistant *E. coli* isolates by PCR [42]. The *aac(6′)-Ib* amplicons were sequenced to identify *aac(6′)-Ib-cr* variant.

The presence of *int*I1 and *int*I2 genes encoding class 1 and class 2 integrases, respectively, and the 3′-conserved segment (3′-CS) (*qac*EΔ1-*sul*1 genes) of class 1 integrons were examined by PCR [42]. Variable regions of class 1 integrons were characterized by PCR and DNA of all *int*I1-positive isolates sequenced [42].

### 4.6. Serotyping and Virulence Genotyping of E. coli

The *E. coli* isolates were screened by single or multiplex PCR assays for the presence of the eight genes encoding the following virulence factors: *fimA* (type 1 fimbriae), *papG* allele III (adhesin PapG class III), *hly*A (hemolysin), *cnf1* (cytotoxic necrotizing factor), *papC* (P fimbriae), *aer* (aerobactin iron uptake system) [encoding virulence factors often found in ExPEC isolates], *eae* (Intimin) and *bfp* (Type IV bundle forming pili) genes [encoding virulence factors often found in EPEC isolates] [43]. EPEC strains possess specific virulence factors such as intimin adhesin (encoded by the *eae* gene) and can be classified as typical or atypical EPEC based on the presence or the absence of bundle-forming pili (encoded by the *bfp* operon) [4]. All isolates were screened for O25a and O25b serotypes [35,44], as well as the new diffuse adhesion encoding *afa* operon (Gen Bank accession number FM955495), specific for O25b:H4 ST131 producing CTXM-15 isolates [44].

### 4.7. Molecular Typing of ESBL-E. coli Isolates

The *E. coli* isolates were characterized by Multilocus-Sequence Typing (MLST) using PCR amplification of the standard seven housekeeping loci [45]. All the amplicons were sequenced and compared with the sequences deposited in the MLST database to know the specific allele combination and the sequence type (ST). In addition, identification of the major phylogenetic groups’ A, B1, B2, or D of *E. coli* isolates was determined by PCR using a combination of three genes (*chuA*, *yjaA*, and *TspE4.C2*), as previously described [46].

### 4.8. Data Analysis

The sequence of extended-spectrum β- lactamase genes and the seven housekeeping genes for each strain were aligned using Muscle (doi:10.1093/nar/gkh340) and Phyml (doi:10.1093/nar/gki352) (1000 bootstrap iterations) software’s to respectively generate the alignment and infer the maximum likelihood-based phylogeny. A model testing was undertaken to assess the use of the GTR model with SMS (https://doi.org/10.1093/molbev/msx149, accessed on 9 September 2017).

The gene presence/absence regarding the antibioresistance and the virulence factors, the phylogroup name, the sequence type, and the pathovar classification were mapped to the phylogenetic tree and visualized with Phandango webtool (doi:10.1093/bioinformatics/btx610).

## 5. Conclusions

Diseased chickens have become an important reservoir of cefotaxime-resistant *E. coli* clones, exhibiting multi-drug phenotypes and harboring various virulence determinants. Our study highlighted the presence of the human high-risk clonal lineages CTX-M-15-B2-O25-ST131 *E*. *coli*, ST10, ST155, and ST58. Despite the low number of studied isolates, ESBL- and AmpC-producing isolates belong to six clones highlighting the general concept of particular clone spread within the considered farm and the risk of circulation of such clones associated with integrons, *qnr* plasmid, and CTXM/CMY genes in healthy animals and humans. In addition, these multi-drug *E. coli* clones were associated with pathogenic pathovars which warrant possible human infections by such strains via direct contact with poultry feces or via the food chain. Hence, increased efforts of antibiotic surveillance to control and regulate the use of antimicrobial agents in poultry farms, in Tunisia, are required to reduce the risk factors associated with the acquisition of multi-resistant clones harboring virulence determinant, allowing prevention and control of the spread of pathogenic bacteria into the environment and to humans.

## Figures and Tables

**Table 1:** Phenotypic and genotypic characteristics of 20 positive cefotaxime-resistant *E. coli,* recovered from fecal and tracheal samples of a diarrheic chicken farm in Tunisia.

Strains	Department	Chicken N°	Swabs	Profiles of Resistance to Non-β-Lactams	Genes Encoding Beta-Lactamases	*bla*_CTX-M_ Genetic Environment	Gene Cassette Arrays in Class 1 Integrons	Other Resistance Genes Detected outside Integrons
EC1	1	C1	T	NAL, SUL, SXT, TET, S	*bla*_CTX-M-15_ + *bla*_OXA-1_	IS*Ecp1/IS26-orf477*	*dfrA17-aadA5*	*tetA, sul2, sul3, aac*(6′)-Ib-*cr*
EC2	1	C1	F	NAL, SUL, TET	*bla*_CTX-M-15_ + *bla*_OXA-1_	IS*Ecp1/IS26-orf477*	ND	*tetA, aac*(6′)-Ib-*cr*, *sul2*
EC3	5	C5	F	NAL, SUL, SXT, TET, S	*bla*_CTX-M-15_ + *bla*_OXA-1_	IS*Ecp1/IS26-orf477*	*dfrA17-aadA5*	*tetA, aac*(6′)-Ib-*cr*, *sul2*
EC4	5	C5	T	NAL, SUL, SXT, TET, S, TOB, CN	*bla*_CTX-M-15_ +*bla*_OXA-1_	Unknown*-orf477*	*dfrA17-aadA5*	*tetA, aac(6′)-Ib-cr, sul2*
EC5	9	C9	T	NAL, CIP, TET, S	*bla*_CTX-M-1_ + *bla*_TEM-1b_	IS*Ecp1-orf477*	ND	*tetB, aadA1, qnrB*
EC6	3	C3	T	NAL, CIP, SUL, SXT, S	*bla*_CTX-M-1_ + *bla*_TEM-1b_	IS*Ecp1-orf477*	*dfrA1-aadA1*	*tetB, qnrB, sul2, sul3*
EC7	9	C9	F	SUL, TET	*bla*_CTX-M-1_ + *bla*_TEM-1b_	*Unknown-orf477*	ND	*tetA*, *sul2*
EC8	12	C12	F	NAL, CIP, SUL, SXT, TET, S	*bla*_CTX-M-1_ + *bla*_TEM-1b_	*Unknown-orf477*	*dfrA1-aadA1*	*tetA, qnrB, sul3*
EC9	6	C6	T	NAL, SUL, SXT, TET, S, C	*bla*_CTX-M-1_ + *bla*_TEM-1b_	IS*Ecp1-orf477*	*dfrA1-aadA1*	*tetA, qnrB, sul2*
EC10	4	C4	F	SUL, TET, S, C	*bla*_CTX-M-1_ + *bla*_TEM-1b_	IS*Ecp1-orf477*	ND	*tetB, sul2*
EC11	4	C4	T	SUL, TET	*bla*_CTX-M-1_ + *bla*_TEM-1b_	IS*Ecp1-orf477*	ND	*tetB, sul2*
EC12	7	C7	F	TET, C	*bla*_CTX-M-1_ + *bla*_TEM-1b_	IS*Ecp1-orf477*	ND	*tetB*
EC13	8	C8	F	SUL, SXT, TET, S	*bla*_CTX-M-15_ + *bla*_OXA-1_ +*bla*_TEM-1b_	IS*Ecp1-orf477*	*dfrA17-aadA5*	*tetA, sul2*
EC 14	8	C8	T	NAL, CIP, SUL, SXT, S	*bla*_CTX-M-15_ + *bla*_OXA1_, *bla*_TEM-1b_	IS*Ecp1-orf477*	*dfrA17-aadA5*	*aac*(6′)-Ib-*cr, sul2*
EC15	6	C6	F	NAL, CIP, S	*bla*_CTX-M-1_ + *bla*_TEM-1b_	IS*Ecp1-orf477*	*dfrA12-aadA2*	*aac*(6′)-Ib*-cr*
EC16	13	C13	F	SUL, SXT, S	*bla*_CTX-M-1_	IS*Ecp1-orf477*	*dfrA12-aadA2*	*sul3*
EC17	2	C2	F	SUL, SXT, S	*bla*_CTX-M-1_	IS*Ecp1-orf477*	*dfrA17*-*aadA5*	
EC18	10	C10	F	SUL, SXT, TET, S	*bla*_CMY-2_ + *bla*_TEM-1b_	IS*Ecp1-*unknown	ND	*tetA, sul2, strA*
EC19	10	C10	T	TET	*bla*_CMY-2_ + *bla*_TEM-1b_	IS*Ecp1-*unknown	ND	*tetA*
EC20	11	C11	T	SUL, SXT, TET	*bla*_CMY-2_ + *bla*_TEM-1b_	IS*Ecp1-*unknown	ND	*tetA, sul2*

EC: *E. coli*; F: Fecal swab; T: Tracheal swab; C: Chicken; NAL: Nalidixic acid; CIP: Ciprofloxacin; SUL: Sulfonamide; SXT: Trimethoprim/Sulfamethoxazole; TET: Tetracycline; C: chloramphenicol; S: Streptomycin; TOB: Tobramycin; CN: Gentamycin; ND: Integron non detected.

**Table 2:** Clonal lineages, pathovars and virulence factor contents in 20-cefotaxime resistance *E. coli* recovered from diarrheic chickens farms.

Strains	Genes Encoding Beta-Lactamases	Virulo-Type */Pathovar	Molecular Typing	
			MLST	Phylogroup
EC1	*bla*_CTX-M-15_ + *bla*_OXA-1_	***cnf1, fimA****, **papG**,aer, **papC***: ExPEC	ST131	B2
EC2	*bla*_CTX-M-15_ + *bla*_OXA-1_	***cnf1, fimA, papG****, aer, **papC:*** ExPEC	ST131	B2
EC3	*bla*_CTX-M-15_ + *bla*_OXA-1_	***cnf1,******fimA, papG,** aer, **papC:*** ExPEC	ST131	B2
EC4	*bla*_CTX-M-15_ + *bla*_OXA-1_	***cnf1,******fimA, papG,** aer, **papC:*** ExPEC	ST131	B2
EC5	*bla*_CTX-M-1_ + *bla*_TEM-1b_	*cnf*, *fimA, **eae***: aEPEC	ST155	D
EC6	*bla*_CTX-M-1_ + *bla*_TEM-1b_	*fimA, cnf1, **eae***: aEPEC	ST58	D
EC7	*bla*_CTX-M-1_ + *bla*_TEM-1b_	***fimA****, **cnf**, aer*: ExPEC	ST155	D
EC8	*bla*_CTX-M-1_ + *bla*_TEM-1b_	*fimA, cnf1, papC, **eae*****:** aEPEC	ST58	D
EC9	*bla*_CTX-M-1_ + *bla*_TEM-1b_	***fimA, cnf1****, aer*: ExPEC	ST155	D
EC10	*bla*_CTX-M-1_ + *bla*_TEM-1b_	*fimA, cnf1, **eae***: aEPEC	ST10	D
EC11	*bla*_CTX-M-1_ + *bla*_TEM-1b_	*cnf1*, *fimA, papC, **eae***: aEPEC	ST10	D
EC12	*bla*_CTX-M-1_ + *bla*_TEM-1b_	*cnf1, fimA, papC, **eae***: aEPEC	ST155	D
EC13	*bla*_CTX-M-15_ + *bla*_OXA-1_ + *bla*_TEM-1b_	***cnf1, fimA, papG***, *hly, **papC**:* ExPEC	ST131	B2
EC 14	*bla*_CTX-M-15_ + *bla*_OXA1_+ *bla*_TEM-1b_	***cnf1, fimA, papG*,***hly, **papC**:* ExPEC	ST131	B2
EC15	*bla*_CTX-M-1_ + *bla*_TEM-1b_	***cnf1, fimA, papC****, hly***:** ExPEC	ST155	D
EC16	*bla*_CTX-M-1_	***cnf1, fimA:*** ExPEC	ST58	A
EC17	*bla*_CTX-M-1_	***cnf1***, ***fimA*:** ExPEC	ST10	A
EC18	*bla*_CMY-2_ + *bla*_TEM-1b_	***cnf1***, ***fimA*:** ExPEC	ST2179	B1
EC19	*bla*_CMY-2_ + *bla*_TEM-1b_	***cnf1***, ***fimA*:** ExPEC	ST2179	B1
EC20	*bla*_CMY-2_ + *bla*_TEM-1b_	***cnf1***, ***fimA*:** ExPEC	ST1011	B1

ST: sequence type; *cnf1*: cytotoxic necrotizing factors; *fimA*: encoding type 1 fimbriae; *papC allele III*: adhesion PapG class III; *papC*: P fimbriae; *eae*: Intimin; *aer*: aerobactin iron uptake system; *hlyA*: hemolysin. *: Virulence associated shown in boldface are the gene characteristics of *E. coli* pathovars.

## Data Availability

All supporting data, code and protocols have been provided within the article or through supplementary data files. One Supplementary Figure S1 is available with the online version of this article.

## Supplementary Materials

The following are available online at https://www.mdpi.com/article/10.3390/antibiotics10060670/s1, Figure S1: Clustering analysis of genetic variation and clonal diversity of 20 ESBL/AmpC producing *E. coli* isolates recovered from fecal and tracheal samples of diarrheic chicken farm in Tunisia. Chicken ID: chicken identified.
