# Supplementary material for: First Detection of Human ST131-CTX-M-15-O25-B2 Clone and High-Risk Clonal Lineages of ESBL/pAmpC-Producing E. coli Isolates from Diarrheic Poultry in Tunisia
Source: Antibiotics (Basel). 2021 Jun 4;10(6):670. doi: 10.3390/antibiotics10060670 (PMC8229138; doi:10.3390/antibiotics10060670)
Supplement: Supplementary file 1 [file antibiotics-10-00670-s001.zip › antibiotics-1149731-supplementary.pdf]

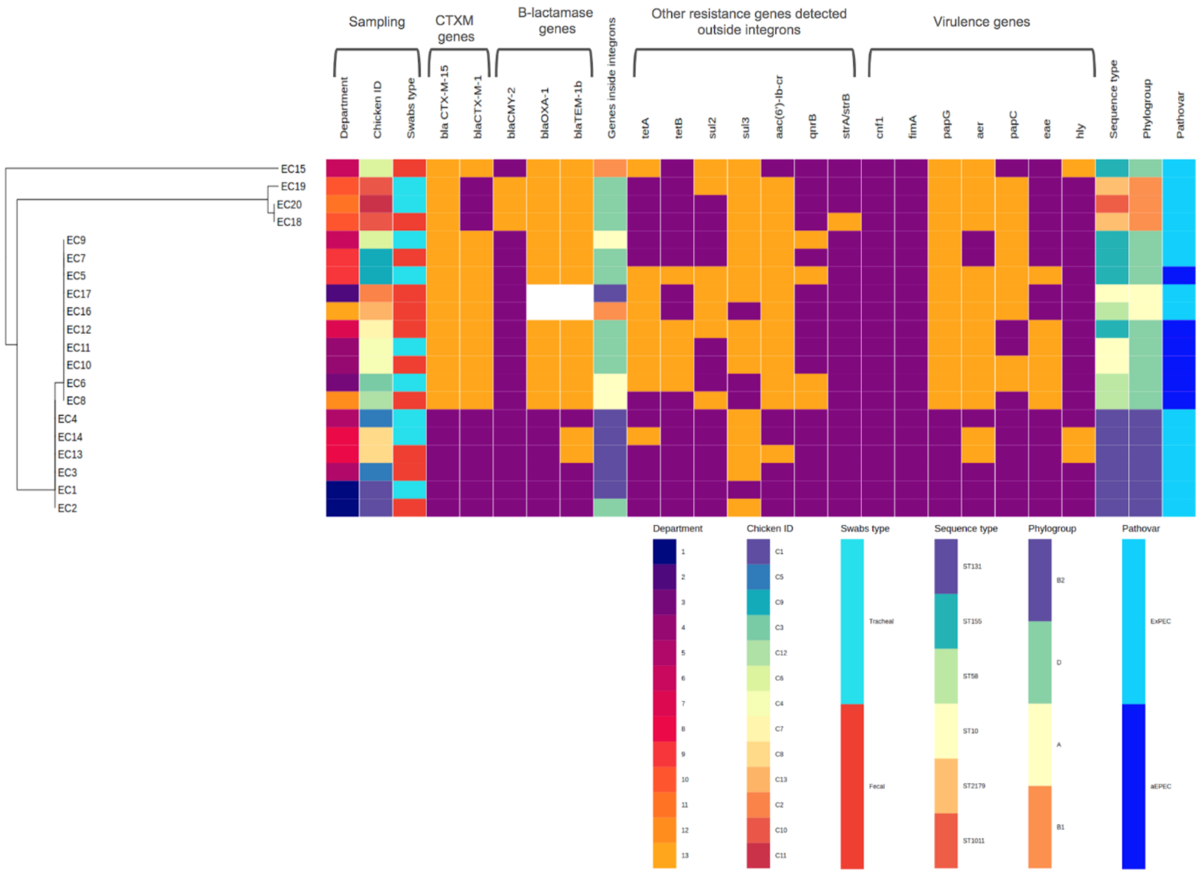

Figure S1. Clustering analysis of genetic variation and clonal diversity of 20 ESBL/AmpC producing *E.coli* isolates recovered from fecal and tracheal samples of diarrheic chicken farm in Tunisia. Chicken ID: chicken identified.
